# The reversal of human phylogeny: *Homo* left Africa as *erectus*, came back as *sapiens sapiens*

**DOI:** 10.1186/s41065-020-00163-9

**Published:** 2020-12-19

**Authors:** Úlfur Árnason, Björn Hallström

**Affiliations:** 1 Department of Brain Surgery, Faculty of Medicine, University of Lund, Lund, Sweden; 2 Center for Translational Genomics, Faculty of Medicine, University of Lund, Lund, Sweden

**Keywords:** Human evolution, Molecular phylogenetics, Progressive phylogenetic analysis, PPA, Palaeontology, Out of Eurasia hypothesis, OOEH, Out of Africa hypothesis, OOAH, mtDNA, nuDNA

## Abstract

**Background:**

The molecular out of Africa hypothesis, OOAH, has been considered as an established fact amid population geneticists for some 25–30 years despite the early concern with it among phylogeneticists with experience beyond that of *Homo*. The palaeontological support for the hypothesis is also questionable, a circumstance that in the light of expanding Eurasian palaeontological knowledge has become accentuated through the last decades.

**Results:**

The direction of evolution in the phylogenetic tree of modern humans (*Homo sapiens sapiens*, *Hss*) was established inter alia by applying progressive phylogenetic analysis to an mtDNA sampling that included a Eurasian, Lund, and the African Mbuti, San and Yoruba. The examination identified the African populations as paraphyletic, thereby compromising the OOAH. The finding, which was consistent with the out of Eurasia hypothesis, OOEH, was corroborated by the mtDNA introgression from *Hss* into *Hsnn* (Neanderthals) that demonstrated the temporal and physical Eurasian coexistence of the two lineages. The results are consistent with the palaeontologically established presence of *H. erectus* in Eurasia, a Eurasian divergence between *H. sapiens* and *H. antecessor* ≈ 850,000 YBP, an *Hs* divergence between *Hss* and *Hsn* (Neanderthals + Denisovans) ≈ 800,000 YBP, an mtDNA introgression from *Hss* into Hsnn* ≈ 500,000 YBP and an Eurasian divergence among the ancestors of extant *Hss* ≈ 250,000 YBP at the exodus of Mbuti/San into Africa.

**Conclusions:**

The present study showed that Eurasia was not the receiver but the donor in *Hss* evolution. The findings that *Homo* left Africa as *erectus* and returned as *sapiens sapiens* constitute a change in the understanding of *Hs* evolution to one that conforms to the extensive Eurasian record of *Hs* palaeontology and archaeology.

## Background

Two molecular studies [1, 2] that came from the same laboratory were highly instrumental in the promotion of the out of Africa hypothesis, OOAH, ≈ 30 years ago. The hypothesis became gradually a mainstay in the discussion of human evolution and dispersal in spite of the absence of palaeontological support for it and the circumstance that reanalysis of the data [3–5] did not favour the phylogenies that constituted the foundation for the OOAH.

The OOAH was addressed previously in two studies [6, 7] in which the author maintained on the basis of molecular and palaeontological data that recent humans, *Hss,* had originated in Eurasia (the Out of Eurasia ypothesis, OOEH) and dispersed from there to other parts of the world including Africa. In the present study the two hypotheses were compared in the light of the identity of the basal divergence of the *Hss* tree as established in genomic analyses [8, 9]. One of these studies [8, Fig. 2a; see also 7, Table 1] identified this divergence as falling between all non-Africans and the African branch of Mbuti/San. The results of the other study [9], which included the Baka population as a representative of basal African lineages and Koinabe (Sahul) as a representative of non-Africans, complied with that finding. The implications of the results were detailed in a previous examination of OOAH and OOEH [7]. As concluded there the reorientation of the *Hss* tree was inconsistent with the understanding that the ancestors of non-Africans had originated by an exodus out of Africa.

The steady progress in the molecular analyses of *Hss* and *Hsn* (*Hsnn* + *Hsnd*) [10–17] has allowed an extended examination of OOAH and OOEH. With the palaeontology of *Hsn* (*Hsnn* + *Hsnd*) strictly limited to Eurasia [18–21] the molecular exchanges between *Hss* and *Hsn* are consistent with a continuous existence in Eurasia of an *Hss* population that is much older than the basal divergence among extant *Hss* populations, i.e. that between non-Africans and Mbuti/San. The palaeontological support for OOEH is upheld similarly by the findings of Eurasian *Hss* fossils [e.g. 22] whose ages, ≈ 200,000 years, surpass by 130,000–140,000 years the age that is commonly associated with the dispersal of *Hss* out of Africa. These finds and the Eurasian placement of the root of the *Hss* tree constitute a natural extension of the extensive Eurasian, palaeontological and archaeological records that relate to the origin and evolution of *Hss* and *Hsn* [23–72]. The existence and hence the phylogenetic implications of these studies have remained disregarded, however, among the proponents of OOAH.

Here the Out of Africa and the Out of Eurasia hypotheses were compared inter alia in the light of a new approach, progressive phylogenetic analysis (PPA), that allowed the examination of the mtDNA relationship among the African *Hss* populations in the light of new palaeontological and molecular findings. The particular result of this examination was that it disrupted the monophyly of the African populations, therewith compromising the cornerstone of OOAH.

## Results and discussion

### The OOEH phylogeny of basal *Hss* relationships

**Fig. 1:**
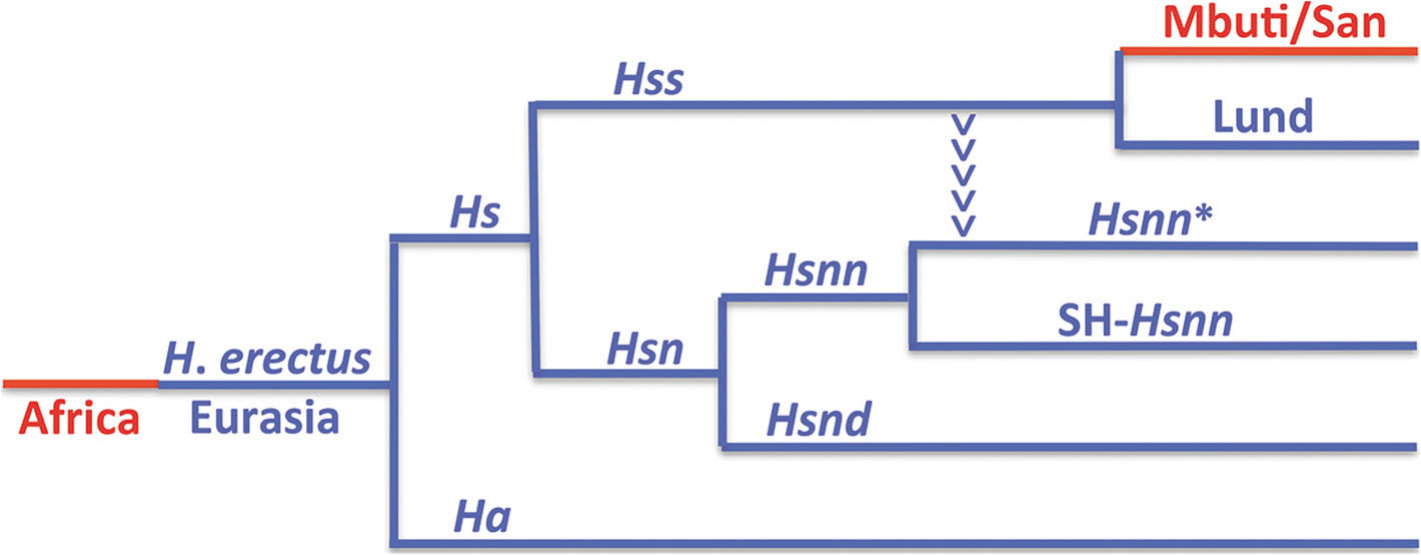
The nuDNA relationship of *Hs*, *Homo sapiens,* and *Ha*, *Homo antecessor*, with Eurasian lineages in blue and African in red. *Hss* (*H. s. sapiens*); *Hsn* (*H. s. neanderthalensis*); *Hsnn* (*H. s. n. neanderthalensis*, Neanderthals proper); *Hsnd* (*H. s. n. denisova*, Denisovans). The divergence between *Hs* and *Ha* has been dated to ≈ 850,000 YBP [73] and that between *Hss* and *Hsn* to ≈ 800,000 YBP. The arrowheads mark the mtDNA introgression that took place from *Hss* into Hsnn* ≈ 500,000 YBP, i.e. more recently than the *Hsnn* divergence into Hsnn* and SH-*Hsnn*. The basal divergence among recent *Hss*, as represented by the Eurasian Lund and the African Mbuti/San is placed in Eurasia at ≈ 250,000 YBP

The essence of the nuDNA phylogeny behind OOEH and the evolution of *Hs* (*Hss* + *Hsn*) is shown in Fig. 1 with the Eurasian lineages marked blue and the African red. The phylogeny encompasses an African exodus of *H. erectus* > 2 MYBP and a continuous evolution of *H. erectus* in Eurasia leading to a population that diverged into *H. antecessor* [66, 73] and *H. sapiens* ≈ 850,000 YBP. The arrowheads that lead from *Hss* into the Hsnn* branch in Fig. 1 have a particular phylogenetic significance for the interpretation of *Hs* evolution as they signify the mtDNA introgression that took place from *Hss* into Hsnn* ≈ 500,000 YBP, a transfer that could only come about in agreement with the physical coexistence of *Hss* and Hsnn*. In the light of the limitation of *Hsnn* to Eurasia the introgression and the continuous evolution of both *Hss* and *Hsn* can only be placed in this continent in accordance with OOEH.

**Fig. 2:**
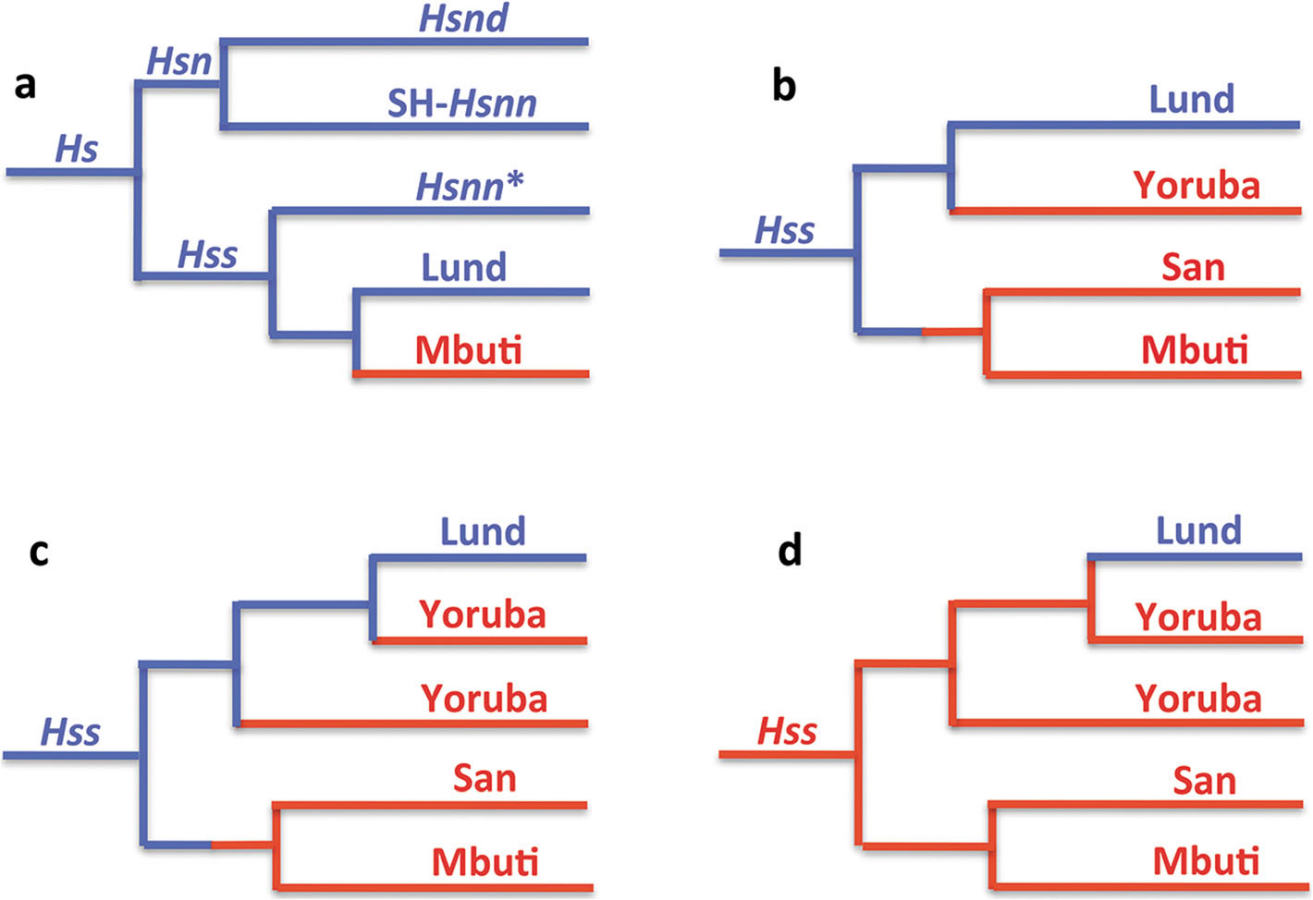
The essence of *Hs* mtDNA relationships as resolved in PPA (Progressive phylogenetic analysis) with Eurasian lineages in blue and African in red. **a**: The *Hs* tree, rooted with the chimpanzee, shows a basal divergence between *Hsn* and *Hss* with *Hsnd* and SH-*Hsnn* on a common branch and Hsnn* joining the *Hss* branch as the result of the mtDNA introgression from *Hss* into Hsnn* ≈ 500,000 YBP. Mbuti and Lund make up the basal divergence among extant *Hss*. **b** and **c**: The inclusion of San and the Yorubas joined San on a common branch with Mbuti, whereas the Yorubas joined the Lund branch at separate positions in accordance with the paraphyly of the African populations. **d**: The OOAH tree with *Hss* origin in Africa followed by a late exodus into Eurasia. The OOAH phylogeny separates Lund from the evolutionary sequence of *Hs* and *Hss* shown in Fig. 1

Figure 2 summarizes the relationships connected to the evolution of *Hs* (*Hss* + *Hsn*) as established in analysis of complete mtDNAs. Tree 2a demonstrates the basal phylogeny of the sampling, while the trees 2b–d show only the *Hss* part of the complete *Hs* tree. The analysis was restricted to the minimum number of taxa required for the examination: a chimpanzee, *Pan troglodytes* [74] for rooting the tree, a Neanderthal proper, *Hsnn* [75], a Denisovan, *Hsnd* [76], Mbuti, San and a series of Yoruba (all African) and Lund, the first described non-chimaeric human mtDNA sequence [74], as a representative of non-Africans. The inclusion of the Yoruba individuals rested upon the circumstance that Yoruba is commonly taken as the African ancestor of non-African populations in studies that acknowledge OOAH [e.g. 77].

Tree 2a shows the phylogeny connected to the arrowhead-marked mtDNA introgression that took place from *Hss* into *Hsnn* as shown in Fig. 1. The introgression, which gave rise to the Hsnn* branch in 2a, upsets the agreement between the nuDNA and mtDNA trees by joining Hsnn* to the *Hss* branch in the mtDNA tree, therewith disrupting the initial mtDNA relationship between Hsnn* and SH-*Hsnn,* an individual representing a population from the Spanish site of Sima de los Huesos, Atapuerca [18–20, 62]. The phylogenetic nature of the arising *Hss* branch is consistent with the strict limitation of *Hsnn*, the mtDNA sister-group of *Hss*, to Eurasia [18–21] and the mtDNA introgression that took place from *Hss* into Hsnn*, the main branch of *Hsnn*, ≈ 500,000 YBP, circumstances that are in accordance with OOEH at the same time as they compromise OOAH.

The *Hss* sampling contributing to tree 2a was restricted to the African Mbuti and the Eurasian Lund, two taxa that represent each of the basal lineages among extant humans. This initial representation was extended by the addition of a San and an initial Yoruba to the PPA sampling in tree 2b. The extension joined San and Mbuti on a common branch while the Yoruba joined Lund on the parallel basal *Hss* branch. In the following PPA step, 2c, the remaining Yorubas were added to the sampling. These Yorubas divided between Lund and the first Yoruba, therewith underlining the separate positions of the Yorubas on the non-African (Lund) branch and the paraphyly of the African *Hss* populations.

The tree in 2d demonstrates the interpretation of *Hss* relationships and evolution in accordance with OOAH. As apparent this tree, with its root placed in Africa and without connection to its sister-group, *Hsn* (*Hsnn* + *Hsnd*), is refuted both phylogenetically and palaeontologically. The tree is similarly incompatible with the basal nuDNA divergence between Mbuti/San and non-Africans [7, 8].

### The phylogenetic signification of the difference between the nuDNA and mtDNA trees of *Hs*

The tree in Fig. 1 shows the relationship of *Hss* and *Hsn* (*Hsnn* + *Hsnd*) consistent with the nuDNA monophyly of *Hss* and *Hsn*. In the light of this relationship the divergence between *Hss* and *Hsn* ≈ 800,000 YBP becomes anchored in Eurasia in accordance with the principle of Last common ancestor and the strict limitation of *Hsn* to this continent as maintained earlier [6, 7] and consistent with the recent *Ha* results [66, 73]. The *Hsn* branch divides into an *Hsnd* branch and an *Hsnn* branch that splits further into SH-*Hsnn* [18–20, 62], and the remaining *Hsnn* [11, 14, 15], labelled Hsnn*. The arrowheads in the figure mark phylogenetically the mtDNA introgression that took place from *Hss* into Hsnn* after the divergence between SH-*Hsnn* and Hsnn*.

**Fig. 3:**
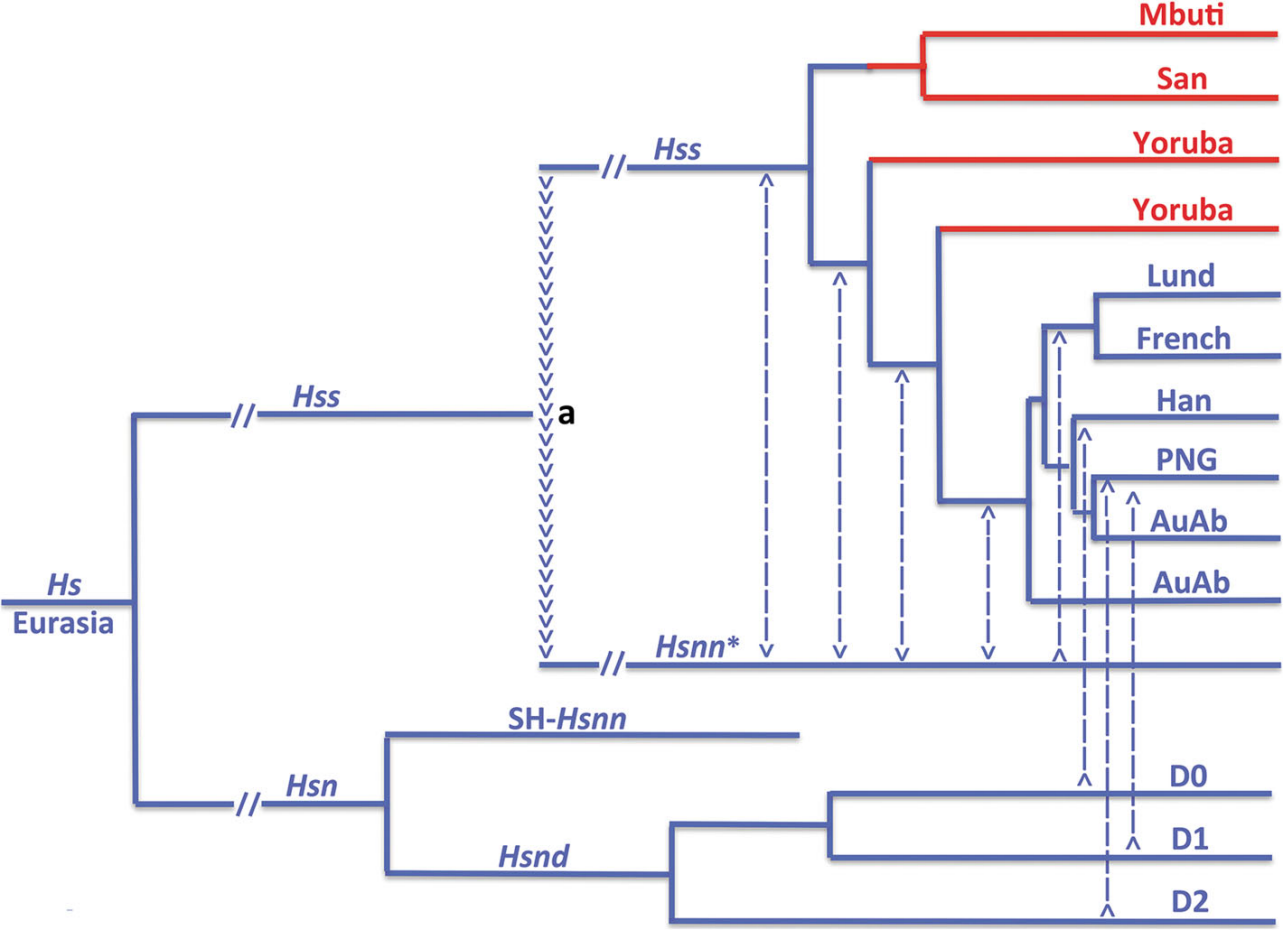
*Hs* mtDNA relationships. Blue: non-African taxa; red: African taxa. The arrowheads (a) signify the mtDNA introgression from *Hss* into Hsnn* ≈ 500,000 YBP, after the *Hsnn* divergence between Hsnn* and SH-*Hsnn* (see Fig. 1). The *Hss* phylogeny underlines the evolutionary continuity leading to the non-African populations and the paraphyly of the African populations [7]. The *Hsn* branch included only one Denisovan in the analysis. Dashed lines indicate general genetic exchanges [11–15] with these from D0, D1 and D2 showing specific input from separate Denisova lineages [78]. The phylogenetic relationship related to SH-*Hsnn* and the Denisovans is in accordance with independent findings [11, 14, 15, 76, 78–80]. AuAb: Australian aborigines; PNG: Papua New Guinean; Han: Chinese; Lund and French: Europeans

The mtDNA tree of *Hs*, Fig. 3, differs fundamentally from the nuDNA phylogeny, Fig. 1, in that *Hsnd* and SH-*Hsnn* have become the sole *Hsn* representatives on a branch (SH-*Hsnn* + *Hsnd*) that is sister to the branch that harbours both *Hss* and Hsnn* as the consequence of the mtDNA introgression that took place from *Hss* into Hsnn* as marked in Fig. 1. The position of the introgression is consistent with a phylogeny that places the transfer after the *Hsnn* divergence between SH-*Hsnn* and Hsnn*. As phylogenetically apparent an introgression in the opposite direction, i.e. from the Hsnn* branch into *Hss*, would join *Hss* and Hsnn* on a branch that was sister to SH-*Hsnn*.

A *Pan*/*Homo* divergence at 8 MYBP [81] places the basal mtDNA divergence in Fig. 3, i.e. that between the branch of (*Hss* + Hsnn*) and that of (*Hsnd* + SH-*Hsnn*) at ≈ 800,000 YBP, the mtDNA introgression from *Hss* into Hsnn* at ≈ 500,000 YBP and the basal divergence among extant humans at ≈ 250,000 YBP. The mtDNA introgression from *Hss* into Hsnn* substantiates the presence of the two lineages in the same area ≈ 300,000 years after the divergence between *Hss* and *Hsn* ≈ 800,000 YBP. With *Hsnn* strictly limited to Eurasia the coexistence of *Hss* and *Hsnn* confirms the restriction of *Hss* to this continent [6, 7]. These phylogenetic, geographic and palaeontological circumstances together with the paraphyly of the African populations as demonstrated by the PPA results and the identity of the divergence between *Ha* and *Hs* are consistent with an evolutionary scenario that anchors the divergence between *Hss* and *Hsn* as well as the mtDNA introgression from *Hss* into Hsnn* in Eurasia, findings that reverse the direction of *Hss* evolution compared to that assumed by OOAH.

**Fig. 4:**
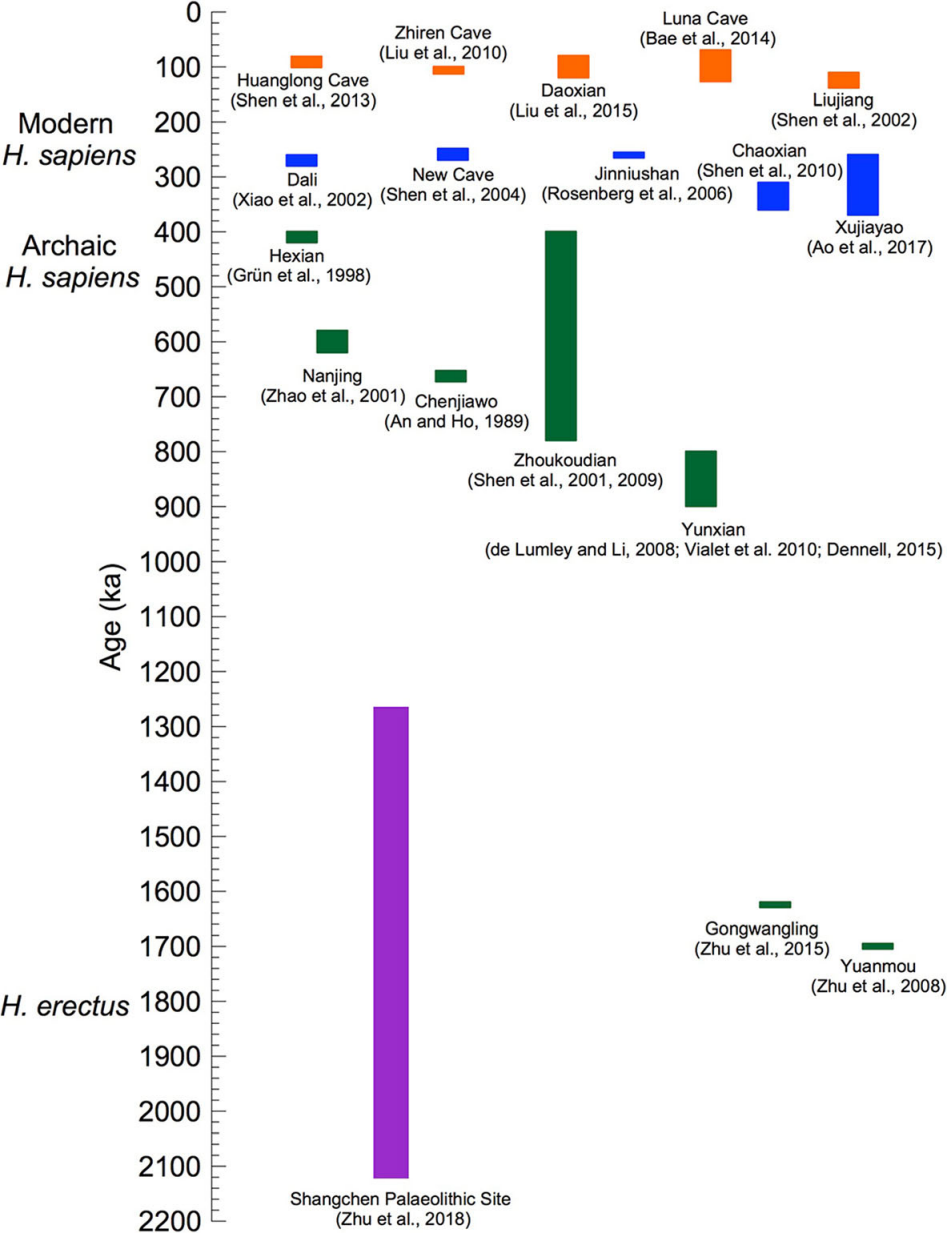
An overview of Chinese palaeontological and archaeological sites related to *Hs* evolution. The Palaeolithic locality at Shangchen (violet) spans the period from 2,12 to 1,26 MYBP. The Yunxian and Zhoukoudian samplings overlap temporally the divergence into *H. antecessor* and *H. sapiens* ≈ 850,000 and *Hss* and *Hsn* ≈ 800,000 YBP, making room for that the two lineages arose from a common Eurasian population of *H. erectus*. The blue section shows more recent *Hss* positions allowing for admixing with *Hsn*(*Hsnn* + *Hsnd*). The same section covers the interval preceding the divergence between Eurasians and Mbuti/San ≈ 250,000 YBP. The Hualongdong fossils [70], discussed in the text, constitute a part of the blue section. The figure has been adapted and extended from Fig. 10 [56], by permission of the authors

The distinction between the mtDNA and nuDNA trees of *Hss* and *Hsnn* was addressed in a molecular study [15] in which the authors related the mtDNA identity of *Hsnn* to an exodus of an African *Hss* lineage into Eurasia and the introgression of the mtDNA of this lineage into *Hsnn*, followed by an extinction of the *Hss* lineage. That scenario is compromised by the phylogenies shown in Figs. 1 and 3 and the extensive and continuous Eurasian palaeontology of both *Hsn* and *Hss* connected to these relationships [e.g. 18–26, 29–34, 38–40, 42–47, 51, 52, 56–58, 60–68]. The age and nature of the *Hss* fossils at Dali [29, 65], New Cave [30], Jinniushan [33] and Xujiayao [56] included in Fig. 4 are of particular interest in this respect as their ages ≈ 250,000–270,000 years underline the Eurasian existence of *Hss* at a time that precedes or coincides with the basal divergence among the ancestors of recent *Hss*.

In addition to the mtDNA introgression from *Hss* into Hsnn* ≈ 500,000 YBP Fig. 3 marks general genetic transfers that took place between *Hsn* (*Hsnn* + *Hsnd*) and different *Hss* populations. The *Hsnd* input into Papuans was addressed in a study [78] that rested upon OOAH and an African *Hss* exodus 64,000 YBP, conclusions that differ from the extension of Eurasian palaeontology. The analysis identified two *Hsnd* introgressions into Papuans, one (D2) at 46,000 YBP the other (D1) at 30,000 YBP. According to the study the D1 branch had split from the Altai Denisovan ≈ 283,000 YBP and the D2 branch ≈ 363,000 YBP. The authors maintained that the D2 estimate was close to that of the divergence between *Hsnn* and *Hsnd*, an underestimate of that particular divergence.

With respect to the basal *Hss* divergence among extant humans there is a fundamental phylogenetic discrepancy between Figs. 1, 2 and 3 and the initial studies advocating OOAH [1, 2], which were based on fragmentary mtDNA data. Reanalysis [3] of the data set behind the first study [1] showed that there were thousands of trees that were better by five steps or more than the tree that constituted the basis of the OOAH argument. The terminology used in the study was also inconclusive as the terms “Europa” and “Caucasians” encompassed Europa proper, North Africa and the Middle East (unspecified). Thus, Africa referred only to a part of this continent, compromising the assumption that “Assuming that Africa is the source, there is only one African cluster”. The phylogenetic problems emerged also in the joining of the HeLa lineage (Afro-American) with one Asian, one Australian Aborigine and one European. Similarly, the composite Cambridge mtDNA sequence [82], with its Afro-American HeLa component, grouped among “Europeans”.

The follow-up study [2] from the same laboratory was limited to parts of the mtDNA control region. The authors maintained that the analysis had yielded the same pattern as the previous study [1] and that the identification of 14 sequential basal African branches provided the strongest support yet for the placement of the human mtDNA ancestor in Africa. As in the case of the first study a reanalysis of the data [4, 5] identified large numbers of trees that were more parsimonious than the tree that the authors [2] presented and based their OOAH reasoning.

### OOEH and OOAH in the light of palaeontology

The fossil record of *Hss* is commonly interpreted in compliance with the preconceptions of OOAH in the discussion of *Hss* evolution. The characterization of the African fossils has a notably wide span, ranging from “The fossil evidence for an African origin for modern humans is robust” [83] to “The hominin fossil record of the African Middle Pleistocene is extremely sparse” [84]. The latter and more inclusive study also detailed that the African fossils were missing adequate provenance in most cases (see Table 1 and Fig. 1 of the study), with the possible exception of the South African Florisbad skull and the age, 260,000 +/− 35,000 years, of the tooth associated with it [85]. Furthermore, Africa contains no fossils of *Hsn* the mtDNA sister group of *Hss*, a crucial phylogenetic circumstance as underlined previously [6, 7]. In comparison the limitation of *Hsn* to Eurasia and the genetic exchanges between *Hsn* and *Hss* provide fundamental palaeontological and molecular results that are consistent with the origin and continuous existence of *Hss* in Eurasia, in accordance with the phylogenies in Figs. 1, 2 and 3.

With respect to the palaeontology and archaeology related to *Hss* evolution it of particular interest to consider the Eurasian advances in this field during the last 20–25 years. The early stages of the progress were discussed in a study [31] that related the morphological skull mosaic between *Hss* and *H. erectus* to a continuous *Hs* admixing in Asia, including gene flow between eastern and more westerly Asia. As regards the morphological distinction between *sapiens* and *erectus* the author [31] recognized them as subspecies, *Homo sapiens sapiens* and *Homo sapiens erectus*, in accordance with their overlapping morphology.

The expansion of Eurasian palaeontology has provided insight into the interwoven evolution of *H. erectus* and *Hss* in Eastern Eurasia [e.g. 23, 26, 29–31, 33, 40, 41, 45, 50, 51, 56, 61, 63, 64, 67, 69, 86]. One of these studies [56] included a figure, modified here as Fig. 4, which showed the chronostratigraphy among a number of Chinese sites that encompassed *H. erectus*, archaic and modern *Hss* and potential *Hsnn* and *Hsnd*. The temporal span of the palaeontological findings extended from 1.7 MYBP to 60,000 YBP. The palaeontological and archaeological site at Xujiayao, which was the primary subject of the study [56], covered the period from ≈ 370,000 YBP to ≈ 250,000 YBP, i.e. a substantial part of *Hss* evolution prior to the *Hss* divergence between Mbuti/San and the branch of other extant humans as shown in Figs. 1, 2 and 3. The Xujiayao site belongs to the particular temporal span (blue) that extends from ≈ 380,000 YBP to ≈ 220,000 YBP in Fig. 4, which the authors [56] related to the late coexistence and genetic admixture of *H. erectus* and early *Hss* plus an *Hsn* input from potentially both *Hsnn* and *Hsnd*. It should be noted that the latter part of this timespan coincides with the genetic exchanges that have been recorded between *Hsnn* and *Hss* before the basal divergence among recent humans [12, 13, 15–17].

The early part of the time range of Xujiayao coincides temporally with the Chaoxian site [40] while the more recent range of the site overlaps with the age of the sites at Dali [29, 65], New Cave [30], Jinniushan [33] and Hualongdong [70], which also provide palaeontological evidence for an extended *Hsn/Hss* interface in Eurasia preceding the basal *Hss* divergence between the ancestors of Mbuti/San and remaining *Hss* populations shown in Figs. 1, 2 and 3. Regarding the volume of the samples from the Xujiayao site it may be noted that they have, in addition to 20 fossil human specimens, yielded more than 30,000 lithic artefacts and about 5000 mammalian fossils that substantiate the nature and age and of the human specimens.

With respect to the age, 1,7 MY, of the Yuanmou finds [36] and that of 1,63 MY of the Gongwangling samples [54] in Fig. 4 it is of crucial interest that their age became exceeded by a wide margin by the artefact sequence from Shangchen, near the Gongwangling site [68]. The 17 continuous artefact layers studied covered a timespan from 2,12 MYBP to 1,26 MYBP. The authors concluded that the findings were in accordance with hominins leaving Africa considerable earlier than indicated by the age, 1,77–1,85 MY, of the *Homo erectus* fossils from the Georgian Dmanisi site [87, 88]. The easterly range of *H. erectus* is represented by the younger Indonesian distribution of the taxon [89–91].

Archaeological sites east of the Mediterranean Sea have revealed a human presence as early as 400,000 YBP [25, 38]. The fossils were not assigned specifically to either *Hss* or *Hsn*, however. In comparison, the human fossils from Qafzeh and Skhul with an age of 90,000–100,000 years were identified as distinct *Hss* [23, 26]. These fossils together with later described *Hss* fossils from the same region, with an age of 180,000–200,000 years [22] fall into the western part of the Eurasian *Hss* distribution that corresponds to the span of modern *H. sapiens* in Fig. 4, i.e. *Hss* after the divergence between Mbuti/San and the remaining Eurasian populations.

The African palaeontology related to *Hs* origin and evolution is distinctly poorer than that of Eurasia. The African *Homo* picture outside *Hss* became extended significantly, however, with the description of the fossils of *H. naledi*, which comprise by far the largest assemblage of *Homo* fossils in Africa. The fossils come from two sites in the South African Rising Star cave system, the Dinaledi Chamber and the Lesedi Chamber [84, 92]. Neither site contains other *Homo* fossils. Dating of the Dinaledi fossils constrains their age to 236,000–335,000 YBP. Another estimate based on the two least-altered *naledi* teeth found yielded a maximum average age of 253 + 82/− 70 thousand years and a minimum average age of 200 + 70/− 61 thousand years [93].

The extensiveness of the *H. naledi* fossils is notable compared to the scarcity of *Hss* fossils in a region that according to proponents of OOAH may have constituted the cradle of *Hss*. Another circumstance relates to the question how *Hss* and *H. naledi*, which might have shared similar ecological niches, could thrive contemporaneously in the same area. Taken together the findings allow the proposal that *naledi* evolved in southern Africa without competition from *Hss* and that it was a later intrusion of *Hss* from the north that led to the demise of *H. naledi*. In this case the Florisbad fossil would constitute a representative of early *Hss* intruders (possibly Mbuti/San) coming from the north.

The redating of the fossils at the Jebel Irhoud site, Morocco [94, 95], which increased their age by ≈ 100,000 years compared to an earlier study of the same site [96], renders the fossils of interest for the discussion of *Hss* evolution in the light of the deepest divergence among recent humans, ≈ 250,000 YBP, and the reversal of the direction of evolution of the *Hs* tree as discussed in connection with Figs. 1, 2 and 3. The age of the tooth examined was 286+/− 32 thousand years, somewhat younger than the age, 315+/− 34 thousand years, of the artifacts connected to layer 7 [94, 95]. The finds have particular implications in the light of the Chinese finds of Dali, New Cave, Jinniushan, Xujiayao and Hualongdong, blue in Fig. 4, which fall in the same part of the temporal interval between the mtDNA introgression from *Hss* into Hsnn* ≈ 500,000 YBP and the basal divergence among recent *Hss* ≈ 250,000 YBP. As the revised age of the Jebel Irhoud fossils exceeds by some margin the age of the basal divergence among extant humans it appears that the Jebel Irhoud population became extinct without contributing to the genetic constitution of extant Africans.

A recent study [97] of the Greek palaeontological finds Apidima 1 and Apidima 2 characterized the Apidima 1 fossil with an allocated age of ≈ 210,000 years as constituting the earliest known presence of *Homo sapiens* (*Hss* here) in Eurasia. This conclusion is implausible considering both the calculation of the age of the Apidima 1 fossil [71, 98] and the large series of much older Eurasian palaeontological finds related to *Hss* evolution that had escaped the attention of the authors [97]. A similar disregard of Eurasian palaeontology appeared in a preceding study [99] that detailed a scenario that rested upon an African origin and divergence between Neanderthals and Denisovans ≈ 480,000 YBP and their exodus into Eurasia ≈ 400,000 followed by an Out of Africa dispersal of modern humans ≈ 75,000–125,000 YBP, circumstances that are incompatible with the extensive Eurasian palaeontology of both *Hsn* and *Hss* and molecular findings, including the molecular exchanges between *Hss* and *Hsn* (*Hsnn* + *Hsnd*). Similarly, a more comprehensive study [100] that appeared in the same journal did not allow a treatment of the Eurasian palaeontology related to *Hss* origin and dispersal as the discussion rested upon OOAH and a temporal demarcation at 125,000 YBP for the oldest Eurasian fossils discussed in the context of *Hss* evolution.

### The OOEH synthesis

The present reorientation of the *Hs* tree in conjunction with the palaeontology of *H. erectus* makes the period ≥900,000 to 500,000 YBP highly significant for the discussion of *Hs* evolution as it covers both the advent and sequel of the divergences that encompass *H. erectus*, *H. antecessor*, *Hss* and *Hsn* (*Hsnn* + *Hsnd*) shown in Fig. 1.

The Asian phase of *H. erectus* evolution ≥900,000 YBP is exemplified in Fig. 4 by the Chinese site at Yunxian [35, 41, 53, 56] and the age, 936,000 years, of the best-preserved scull (Yunxian II) excavated at this site [35, 41]. With regard to the Yunxian II specimen one of these studies [41] concluded that the facial features of the fossil displayed a pattern close to modern humans and that the assignment of the scull to *H. erectus* extended the variability connected to this species. In this light and in recognition of the fossil record of *H. erectus* the findings are consistent with the origin of a branch that arose within a diversifying Asian *H. erectus* population which diverged into the westward going population of *H*. *antecessor*, which came to reside for a short period in SW Europa, and another population, *H. sapiens*, that split later into *Hss* and *Hsn*. According to this understanding *Hsn* diverged further into *Hsnd* and *Hsnn* with *Hsnd* diversifying essentially in eastern Asia and the Sahul and *Hsnn* inhabiting a large Eurasian area within which it diversified into a westerly branch, SH-*Hsnn*, and a more widely spread population, Hsnn*, characterized by the mtDNA introgression from *Hss* ≈ 500,000 YBP, a circumstance that manifests the lasting and contemporary Eurasian presence of *Hss* and *Hsnn*.

It should be noted that the *Hss* part of the mtDNA tree in Fig. 3 suggests that the Eurasian *Hss* population went through a severe bottleneck prior to the diversification shown in the figure. In comparison the African population structure suggests that the populations arising from the different *Hss* exoduses into Africa remained relatively unaffected in this respect.

### *Hss* evolution in relation to climatic changes

The estimated ages of the divergences related to *Hss* and *Hsn* show considerable variation among authors depending on the calibration points chosen. In the present study we have applied as a calibration point the *Homo/Pan* divergence set at 8 MYBP, in accordance with the mammalian calibration point A/C-60 [81]. As mentioned above the 8 MY age of the *Homo/Pan* divergence places the basal *Hs* divergence between *Hss* and *Hsn* at ≈ 800,000 YBP and the mtDNA introgression from *Hss* into Hsnn* at ≈ 500,000 YBP. Within the *Hss* lineage itself the basal divergence, that between Mbuti/San and other recent lineages, becomes placed at ≈ 250,000 YBP, the time of the two Yoruba exoduses into Africa at ≈ 225,000 and ≈ 180,000 YBP respectively and the age of the basal divergence among recent non-Africans at ≈ 125,000 YBP. The ages of three of these estimates, ≈ 250,000, ≈ 225,000 and ≈ 125,000 YBP, coincide with warm global temperatures that were preceded by low maxima as recorded in analyses of Antarctic ice core records [101–103]. The picture is consistent with a Eurasian *Hss* population that passed three cold-related bottlenecks, each of which was followed by population expansion and concomitant dispersal. Although the coincidence between the timings of the molecular estimates and climatic changes might not be absolute they underline the scenario of oscillating climatic conditions that are likely to have affected vegetation and population structures among both humans and their prey in connection with changes in sea levels and routes of dispersal.

The molecular variation within Africa in both mtDNA [104] and nuDNA [8] is greater than that among non-African populations. Among population geneticists this variation has been commonly interpreted as supporting OOAH without considering the phylogenetic nature of the relationships connected to the different *Hs* divergences leading to recent humans. The identity of these relationships as maintained here and the paraphyly of the African populations suggests a different explanation, namely that the African variation is the effect of consecutive *Hss* dispersals from Eurasia into Africa and climate-related Eurasian *Hss* bottlenecks that reduced the non-African variation, the most distinct being that ending ≈ 125,000 YBP. A scenario of this kind concurs with climate history and the glaciation cycles discussed above [101–103]. It has been noted similarly in several studies [17, 105–107] that the amount of *Hsnn* DNA in African genomes is less than that of non-Africans, a finding that is consistent with the African absence of both Neanderthals and Denisovans and an extended contact by Neanderthals and Denisovans with the non-African populations.

**Fig. 5:**
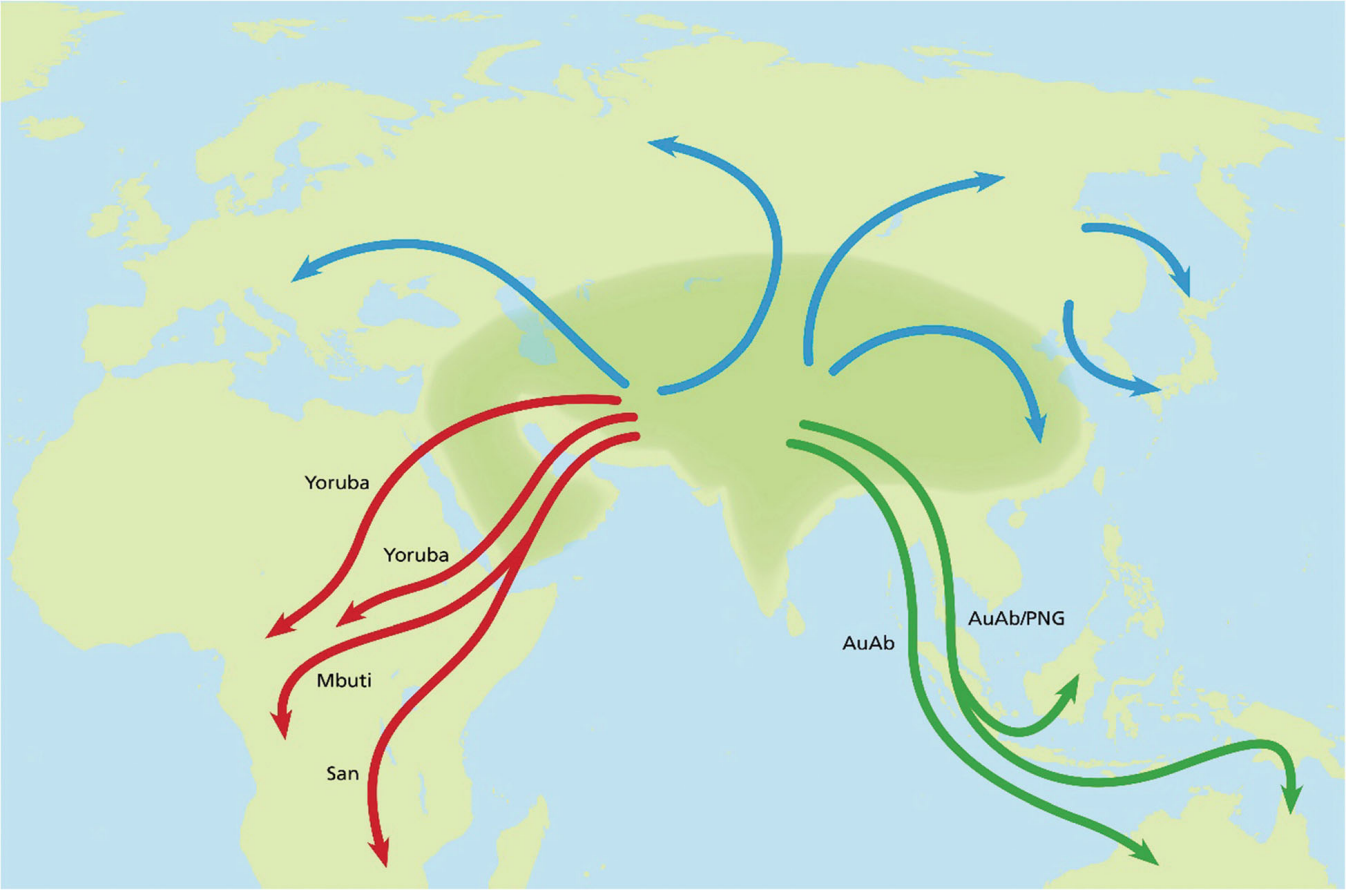
A simplified view of *Hss* dispersal. The shaded area signifies a geographically undefined Asian (Eurasian) area from which *Hss* dispersed. Mbuti/San mark the earliest *Hss* exodus into Africa followed by later Yoruba exoduses. The green tracks represent routes that signify potential routes into Southeast Asia and Oceania

Figure 5 shows potential routes related to the dispersal of *Hss* in accordance with the PPA findings and the relationships behind Figs. 1, 2 and 3. The depicted routes allow ample room for the genetic exchanges between *Hss* and *Hsnn* and *Hss* and *Hsnd* in Eurasia and more south-easterly regions that have been detailed in a series of nuDNA studies [e.g. 10, 12, 13, 15–17, 80, 81, 108–111].

## Conclusions

The reversal of the direction of evolution in the tree of *Homo sapiens sapiens* is consistent with the molecular identification of the sister group relationship between *H. sapiens* and *H*. *antecessor* and a Eurasian separation into *H. antecessor* and *H. sapiens* (*Hss* + *Hsn*) ≈ 850.000 YBP [66, 73] within a diversifying population of *H. erectus*. The hitherto earliest recorded phase of hominin presence in Eurasia, that at Shangchen, China, has been dated to ≈ 2.1 MYBP [68]. The finds constitute a temporal extension of the palaeontological findings of *H. erectus* in Dmanisi, Georgia, ≥ 1.8 MYBP [87, 88] and Yuanmou, China, 1.7 MYBP [36] and a series of younger finds that underline the Eurasian existence of *H. erectus* preceding the divergence between *H. antecessor* and *H. sapiens*.

We postulate that following the separation between *H. sapiens* and *H. antecessor* ≈ 850.000, *Hs* diverged further into *Hss* and *Hsn* ≈ 800.000 YBP. The Eurasian continuity of these two lineages is underlined by the molecular diversity of *Hsnn* and *Hsnd* and the mtDNA introgression that took place from *Hss* into Hsnn* ≈ 500,000 YBP. In comparison the *Hss* branch remains undivided until the basal divergence among the ancestors of extant *Hss* as represented in Fig. 1 by Lund and the African populations of Mbuti/San, therewith bringing to an end the evolutionary journey that began by *Homo erectus* leaving Africa ≥2 million years earlier.

## Appendix

OOAH has been assumed as a phylogenetic fact for some 30 years in the molecular discussion of *Hs* (*Hss* + *Hsn*(*Hsnn* + *Hsnd*)) evolution in spite of the absence of palaeontological support for the hypothesis. A concerning circumstance related to the hypothesis is that the initial mtDNA studies [1, 2] reported phylogenies that were inconsistent with a large series of better trees [3–5], a condition that compromised the phylogenetic conclusions of both studies. Unfortunately, the authors [1, 2] did not clarify or respond to this crucial issue, therewith promoting the impression that the criticism [3–5] was unsubstantiated.

The survival of OOAH draws attention to a particular case at the Institute of Genetics, University of Lund, our previous premises, that terminated the long–lasting belief in the erroneous human chromosome number, 2n = 48. The case relates to the establishment of the human karyotype and the correct chromosome number, 2n = 46, under the auspices of Prof. Albert Levan [112] as detailed at the 50 year anniversary of the findings [113]. The study in question [112] rested upon the introduction of three essential cytogenetic legs, viz. the use of cell cultures (delivered by Dr. Rune Grubb providing dividing single cells), the application of colchicine (for constricting the chromosomes and arresting the cells in metaphase), and hypotonic treatment (for the swelling of the cells prior to fixation). The work [112] paved the way for an explosive expansion of cytogenetics at the same time as it dispelled an understanding that had reigned for more than 40 years with the last 2n = 48 count becoming that by Prof. C. D. Darlington and his collaborator who reported this number in two males [114].

In 2006, at the 50 years anniversary of the study of Tjio and Levan [112], there appeared a few accounts that presented diverging details related to the establishment of the 2n = 46 number. The major discrepancies among these descriptions could be resolved, however, by the fortunate finding of the logbook of Levan’s laboratory [113].

**Fig. 6:**
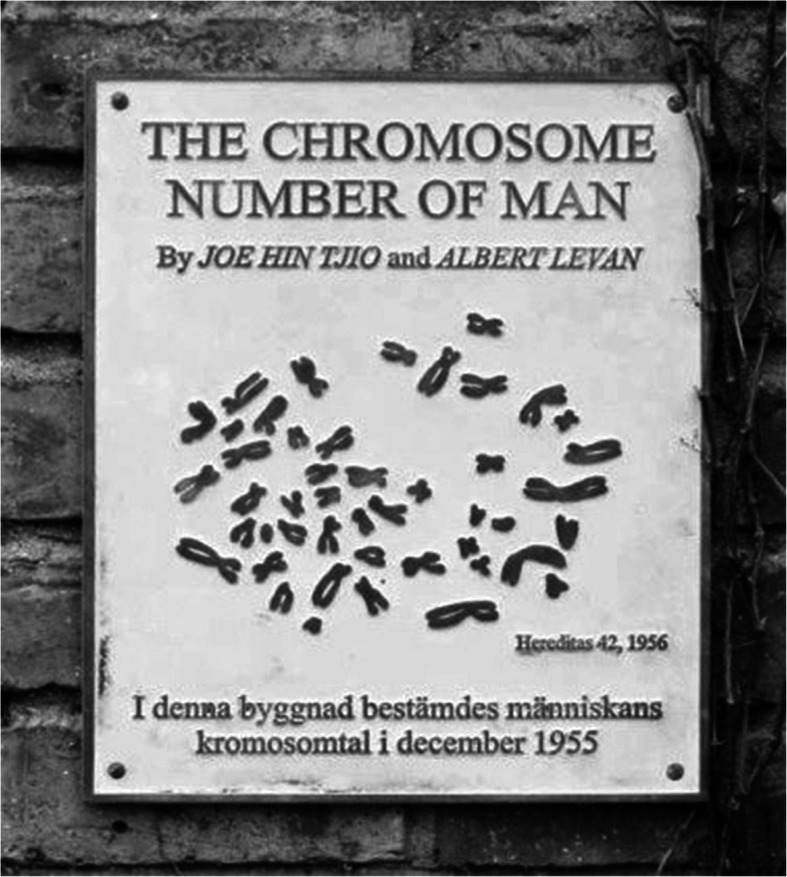
The no longer extant copper plate commemorating the establishment of the 2n = 46 chromosome number of man at the Institute of Genetics, University of Lund, Sweden

A copper plate, Fig. 6, commemorating the establishment of the 2n = 46 human karyotype was uncovered at the Institute of Genetics on March 8th, 2003, on the 100th birthday of Karin Levan, Albert Levan’s wife. The title of the paper, the names of the authors and the incorporated metaphase are replicates from the original publication [112]. The Swedish text reads in translation: “The chromosome number of man was determined in this building in December 1955”. The plate became stolen later. It has not been replaced and the discovery has fallen into oblivion.

## Data Availability

The datasets generated during and/or analyzed during the current study are available from the corresponding author on reasonable request.

## Abbreviations

*Ha*: *Homo antecessor*
*He*: *Homo erectus*
*Hs*: *Homo sapiens*
*Hss*: *Homo sapiens sapiens*
*Hsn*: *Homo sapiens neanderthalensis* (Neanderthals + Denisovans)
*Hsnn*: Neanderthals proper
SH-*Hsnn*: Neanderthals from the Spanish site of Sima de los Huesos representing a branch that evolved without the introgression of *Hss* mtDNA
*Hsnn**: Neanderthals characterized by the mtDNA introgression from *Hss*
*Hsnd*: Denisovans
